# Somatic Stem Cells: Control of Quiescence Versus Activation in Aging and Cancer

**DOI:** 10.3390/cancers18162542

**Published:** 2026-08-07

**Authors:** Daniel Yi, Michael Kahn

**Affiliations:** Cancer Biology and Molecular Medicine, Beckman Research Institute, Duarte, CA 91010, USA; daniel.yi.hz@gmail.com

**Keywords:** CBP, p300, beta-catenin, stem cell, quiescence, enhancer, super-enhancer, ICG-001, cancer stem cell

## Abstract

Somatic stem cells, as well as cancer stem cells, exist in two basic “states”, quiescent versus activated. The regulation of the balance between quiescence and activation is critical to tissue homeostasis and repair after injury. Additionally, after being activated, the decision to divide symmetrically or asymmetrically is critical. Aberrant regulation of stem cell quiescence and mode of mitotic division is associated with aging and diseases of aging including cancer, fibrosis, sarcopenia and neurodegeneration. Based on over 25 years of chemical genetic and genetic investigation, we discuss the differential roles of the two Kat3 coactivators (*Kat3A/CREBBP*/CBP and *Kat3B/EP300*/p300) in regulating stem cell quiescence and the mode of division of activated stem cells. The ability to pharmacologically regulate differential Kat3 coactivator usage has important implications to ameliorate the aging process as well as to eliminate quiescent cancer stem cells, which are the cause of disease relapse and metastasis.

## Simple Summary

Somatic stem cells are responsible for both homeostatic processes as well as repair after injury of our bodies. They exist in two basic states, quiescent or activated. A similar situation exists for cancer stem cells. Based on more than twenty-five years of research, we propose that the key to controlling the balance between quiescence and activation lies in differential usage of the Kat3 transcriptional coactivators CBP and p300. The ability to pharmacologically regulate differential Kat3 coactivator usage has important implications to ameliorate the aging process as well as to eliminate quiescent cancer stem cells, which are the cause of disease relapse and metastasis.

## 1. Introduction

**Figure 1:**
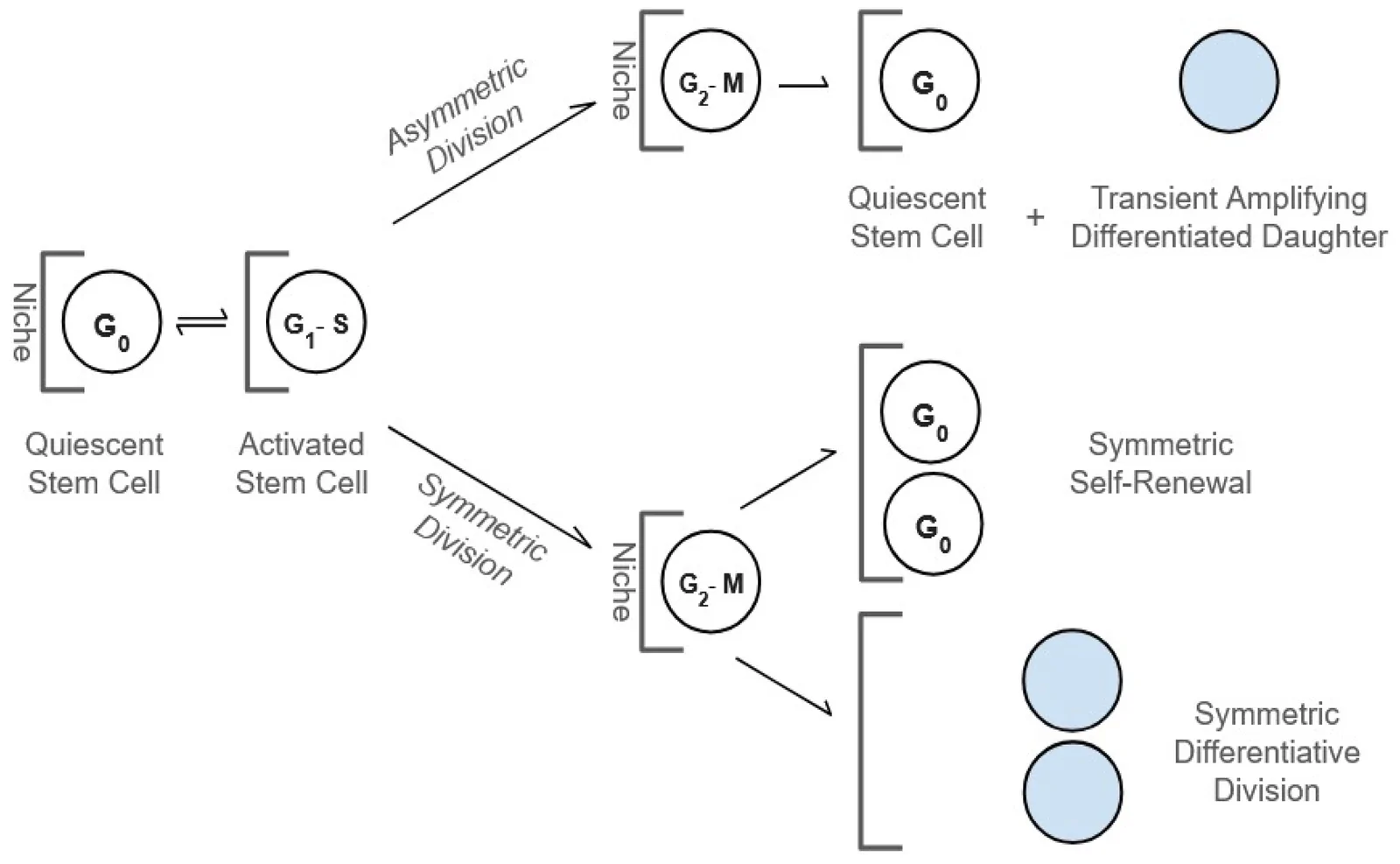
Asymmetric division results in the production of two daughter cells with different cell fates—one a stem cell and the other a differentiated transient amplifying (TA) cell (**upper**). A symmetric non-differentiative division occurs when the two daughter cells remain as stem cells. A symmetric differentiative division gives rise to two daughter cells, both of which are differentiated TA cells (**lower**).

The term somatic stem cell covers a very broad range of highly heterogeneous cell types. Yet they all exist in two basic “states”, quiescent versus activated. Once activated, they have a critical decision to make, i.e., to divide symmetrically or asymmetrically. Thus, it would appear at first glance that somatic stem cells—which live with us our entire lives and are responsible for both tissue homeostasis and repair—have a relatively uncomplicated decision tree (Figure 1).

However, these apparently simple decisions are not arrived at trivially. The ability to re-enter the cell cycle upon receiving the proper cues defines the transition from stem cell quiescence to activation and sets up the next critical decision, i.e., mode of division. Additionally, activated stem cells may need to readopt their quiescent status to prevent premature differentiation and exhaustion. Therefore, not surprisingly, aberrant regulation of stem cell quiescence and mode of mitotic division is associated with aging and diseases of aging including cancer, fibrosis, sarcopenia and neurodegeneration [1,2,3,4]. In this Insight, based upon more than 25 years of genetic and chemical genetic investigations, we discuss what we have learned regarding the roles of the two Kat3 coactivators (i.e., *Kat3A/CREBBP*/CBP and *Kat3B/EP300*/p300) in regulating stem cell quiescence and the mode of division in stem cells.

## 2. Quiescence

Quiescence is a feature common to both normal somatic stem cells (SSCs) and cancer stem cells (CSCs). During our lifetime, SSCs are required for both repair after injury and homeostatic regeneration; however, SSC functionality declines with aging [5,6,7]. Quiescence and asymmetric division assist SSCs by minimizing damage to their DNA, organelles, lipids and membranes, to maintain their long-term fidelity and functionality [8,9,10]. The “dark-side” of quiescence is that quiescent CSCs constitute a reservoir associated with disease resistance and, when reactivated, disease relapse [11]. To affect a true cancer “cure”, the elimination of quiescent CSCs is essential [3,12,13,14,15].

## 3. Quiescence and Aging

Quiescence requires the integration of a complex set of cell intrinsic and niche factors. However, the fidelity of exit from quiescence declines with age. Quiescence and subsequent asymmetric division of activated somatic stem cells are critical in the protection of our essentially immortal SSCs from damage incurred by DNA replication and metabolic activities [16]. Based, in large part, on transbiosis experiments, the problem seems to be primarily extrinsic and less an intrinsic defect in aged SSCs [10]. It has been demonstrated that circulating factors can accelerate aging in young organisms exposed to old blood or induce rejuvenation in old organisms exposed to young blood [17]. Therefore, in principle, we should be able to “rejuvenate” aged endogenous stem cells utilizing extrinsic therapeutic agents.

## 4. Quiescence Regulation and Mode of Division

Although simple in principle, the decision for a stem cell to remain quiescent or enter mitosis requires a complex integration of niche microenvironmental signals, including oxygen, nutrient and metabolite levels, circadian rhythms, neural innervation, adhesion molecules, and key developmental pathways such as Wnt, Notch, Hedgehog, and TGF-β [1,3,10,16,18]. Activated mitotic stem cells, whether SSCs or CSCs, integrate this enormously complex array of information to arrive at the decision to remain quiescent or once activated to divide asymmetrically or symmetrically [10].

Long-lived SSCs preferentially undergo asymmetric versus symmetric cell divisions. The rationale for this was outlined more than 40 years ago in the “immortal strand hypothesis” [19,20,21,22]. Cairns posited that SSCs undergo mitosis, wherein the “mother” stem cell retains the original strands of DNA and the differentiated daughter cell inherits the duplicated strands containing multiple copy errors, inherent in the DNA replication process. Thereby, accumulating mutations in long-lived SSCs can be minimized [19,20,23,24]. Importantly, a fundamental difference between CSCs and normal SSCs is that CSCs often exhibit a preferential shift toward symmetric self-renewal under specific oncogenic and microenvironmental contexts.

For example, p53 mutated breast cancer stem cells preferentially undergo symmetric divisions [25]. In normal hematopoietic stem cells (HSCs), PTEN loss of function (LOF) leads to premature exhaustion (due to increased symmetric differentiative divisions), whereas the leukemic stem cell (LSC) population increases (due to increased symmetric non-differentiative divisions) [26]. This is also a feature associated with premalignant stem cells, for example, in Clonal Hematopoiesis of Indeterminate Potential (CHIP) [27]. However, CSCs can also undergo asymmetric division, leading to increased intra-tumoral heterogeneity [28]. Although the immortal strand hypothesis is still debated [23,24,29,30], it has become clear that asymmetric partitioning of stem cell DNA, organelles (e.g., mitochondria, lysosomes, centrioles), lipids, proteins (e.g., histones, β-catenin) RNA and membranes are critical aspects of asymmetric stem cell divisions [31,32,33].

## 5. Immune Cell Quiescence and Memory

Quiescence and activation in T cells are critical for an effective immune response to vaccination and infection, while preventing inappropriate responses, for example, in autoimmunity. Quiescence in T cells is regulated by many of the same factors discussed above that regulate SSCs. With aging, loss of quiescence in T cells is associated with a limited immune response to infection or vaccination [34]. Older individuals are more prone to morbidity and mortality after viral infection and have poorer memory responses to vaccination. The recent COVID-19 pandemic and seasonal influenza outbreaks confirmed that older individuals are at a disproportionate risk for severe disease [35,36,37]. The transcription factor FOXO1 is important for maintaining quiescence and the expression of FOXO1 decreases with age. This is associated with the exhaustion of stem cells and the terminal differentiation of T cells [38]. Asymmetric cell division is also a critical early step in T lymphocyte fate divergence, giving rise to daughter cells that either terminally differentiate to effectors or self-renewing memory lineages. This is controlled by cell-intrinsic and -extrinsic cues from the microenvironment [39]. Asymmetric lymphocyte differentiation also deteriorates with aging [40,41].

## 6. Kat3 Coactivators; “Nothing in Biology Makes Sense Except in the Light of Evolution”—Theodosius Dobzhansky

**Figure 2:**
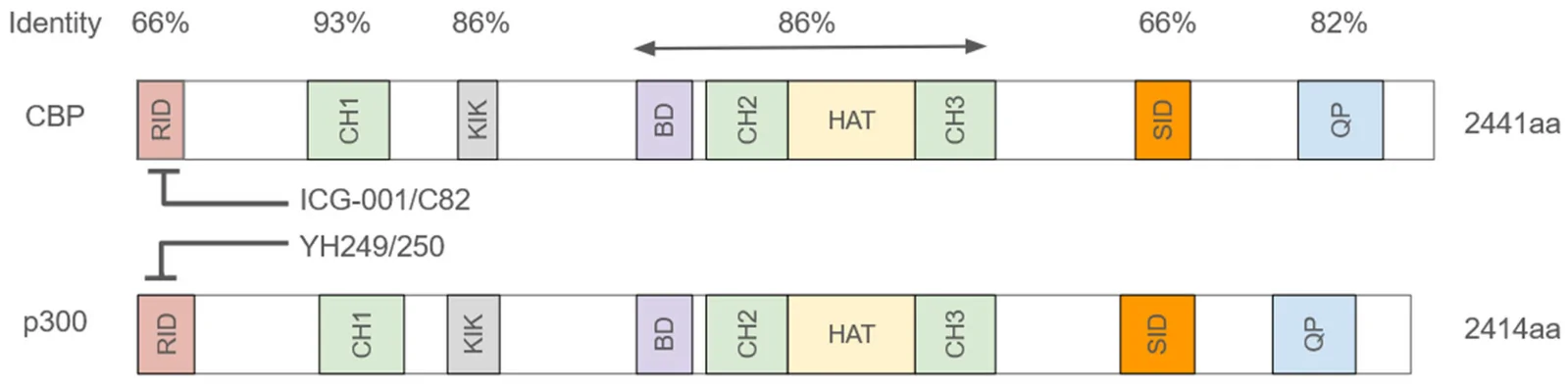
Schematic depicting CBP and p300 and the high percentage of identity at the amino acid level between various regions of these large Kat3 coactivators, despite their divergence more than 450 million years ago. The very amino terminal of CBP, to which both CBP/beta-catenin (ICG-001 and C82, the active agent derived from the pro-drug PRI-724) and p300/beta-catenin antagonists (YH249/250) bind, is by far the most divergent region between these two Kat3 coactivators.

The critical question to address then is, “what is the molecular mechanism utilized to distill down and integrate this plethora of information into the decision to exit quiescence and subsequently to undergo a symmetric or asymmetric division?” Vertebrates began their evolution approximately 450 million years ago, initiating a new lifestyle with a complex body plan and a relatively long-lived adult stage. This “lifestyle” necessitated long-term homeostatic stem cell maintenance for tissue renewal and repair. To accomplish this, SSCs in their corresponding niches had to maintain a relatively quiescent “anaerobic” metabolic state, as opposed to their more proliferative aerobic-differentiated daughter cells, to protect the integrity of the genetic material in SSCs [42]. A high-fidelity mechanism ensuring asymmetric SSC division, with the maintenance of “stemness” in the “mother” cell and the generation of a differentiated rapidly dividing daughter cell, was required. The evolution of the Kat3 coactivator family, *Kat3A/CREBBP* (CBP) and *Kat3B/EP300* (p300), which diverged via a gene duplication over 450 million years ago, just prior to the vertebrate radiation, provided a solution to this problem [10,43]. They are both very large proteins with molecular weights of ~300 kd, encoded over 33 and 31 exons respectively. Yet they retain an extremely high degree of identity, up to 93%, in particular, over a large central core including the CH1, KIX, Bromodomain, and CH2 and CH3 regions (Figure 2) [44]. As master orchestrators at enhancers (E) and super-enhancers (SE), CBP and p300 each can interact with more than 400 proteins. Due to their high degree of protein sequence identity and similar roles, perhaps not totally unsurprisingly, they have long been and still are often considered largely redundant [45]. However, it is now clear that CBP and p300 are not redundant and have unique roles both in vitro and in vivo [3,10,46,47,48,49,50].

Furthermore, “Mother Nature” does not conserve such large genes on two different chromosomes for more than 450 million years without a very good reason! The N-termini of CBP and p300, to which the small molecule CBP (ICG-001 and C82, the active agent derived from dephosphorylation of PRI-724) and p300 (YH 249/250)/β-catenin antagonists, bind, are by far the most divergent region between the two Kat3 coactivators, with only 66% identity. The N-terminal regions, to which β-catenin binds, also contain a highly conserved LXXLL sequence, a short, conserved amino acid motif found in coactivator proteins that acts as a “signature” for binding to activated nuclear receptors (NRs) [51] and a Stat1/2 binding site [52]. Thereby, the N-term can serve as a signaling nexus for activation/proliferation/differentiation, metabolism and the immune response. Evolution optimized these two signaling hubs in the N-terminal regions.

However, once optimized, the sequences within each orthologous group have been extremely highly conserved for at least the past 100 million years (i.e., human and mouse CBP are 98% identical at the amino acid level within this region of each protein) [43]. The requirement for long-term homeostatic stem cell maintenance in vertebrates drove the rapid divergence, and the very high degree of conservation once optimized, of the two Kat3 N-terminal regions [10].

## 7. Aging Stem Cells

With aging, contrary to earlier beliefs, the number of SSCs increases; however, their efficiency decreases [4,9,10,34,36,37]. This is associated with an increase in the number of symmetric self-renewing divisions at the expense of asymmetric divisions in SSCs along with increased SSC quiescence [9,10]. Consistent with this, a very recent report showed that the number of neural stem cells (NSCs) was significantly increased in adults with preclinical cognitive decline and Alzheimer’s Disease (AD), compared to normal for their age cognitively healthy adults (HAs). Additionally, the average number of neuroblasts and immature neurons formed from differentiating NSCs was significantly reduced in the AD group compared with HAs and young adults [53]. Based on our previous studies, this block in NSC differentiation may be correctable using a CBP/β-catenin antagonist [54,55].

Multiple factors, including mutations, diet and inflammation, can affect SSC quiescence and the mode of division of activated SSC [1]. Mutations in the *LMNA* gene are associated with Hutchinson–Gilford progeria syndrome (HGPS). These mutations, which cause accelerated aging, compromise nuclear structure, induce persistent DNA damage and decrease asymmetric stem cell division [56]. Diets including added sugars, sodium, and processed meats, high in ultra-processed foods and low in whole, plant-based foods can accelerate aging [57]. Chronic, low-grade inflammation—often termed “inflammaging”—is a key driver of accelerated aging, by damaging tissues, accelerating cellular senescence and decreasing the fidelity of the SSC pool [58]. Inflammaging, cellular senescence, and the Senescence-Associated Secretory Phenotype (SASP) are interconnected processes that drive aging and age-related diseases [59].

These all appear to be associated with an increase in CBP/β-catenin-driven transcription at the expense of p300/β-catenin-driven transcription with aging and are consistent with epidemiologic data that the risk of developing cancer, fibrosis, or neurodegeneration increases significantly around age 50 [1,3,10,60].

## 8. Implications

**Figure 3:**
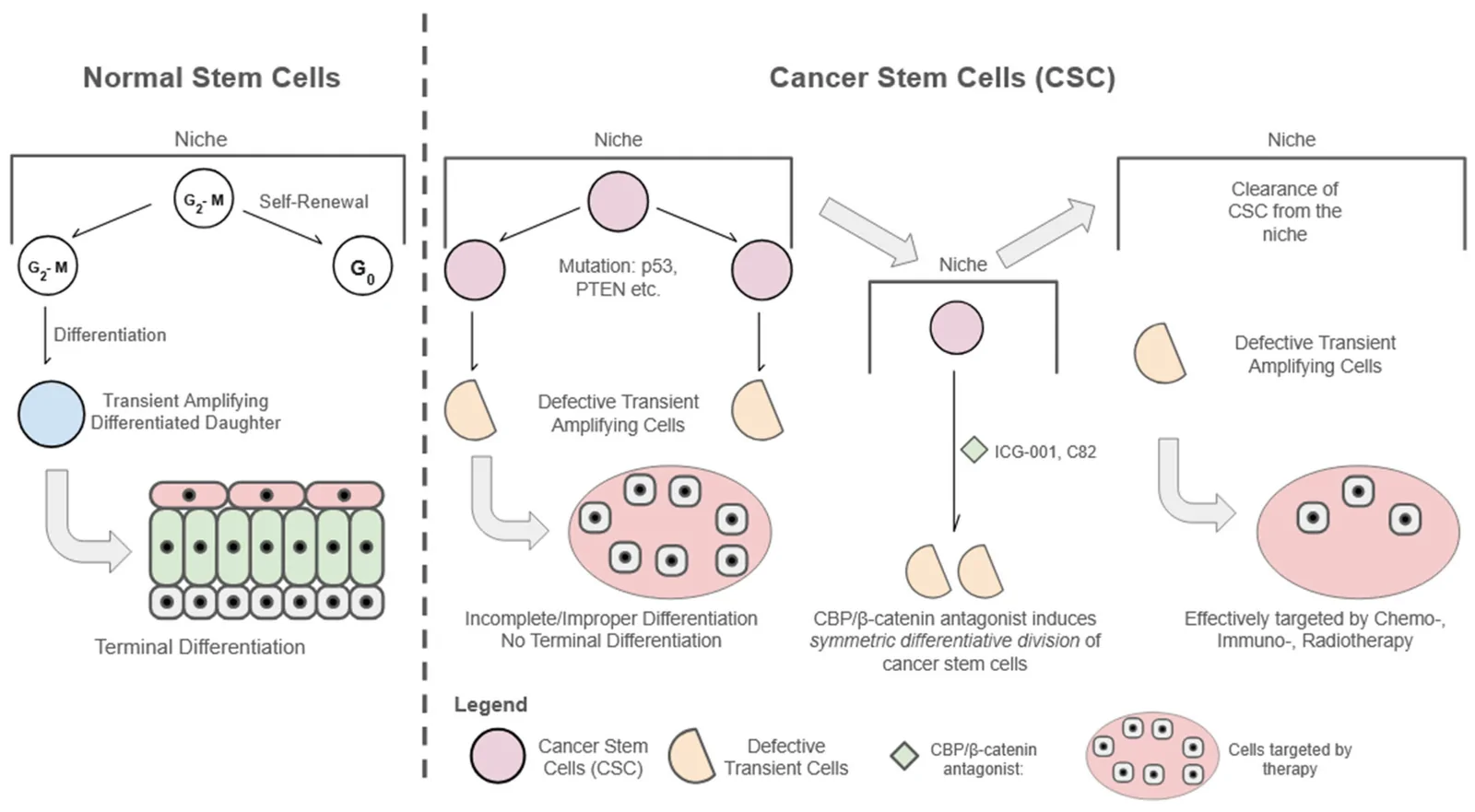
In this model, normal somatic stem cells (SSCs), asymmetric division is favored, wherein one daughter remains in the niche as a stem cell and the other transiently amplifies and differentiates to generate new tissue (**left panel**). Cancer stem cells (CSCs) undergo both symmetric and asymmetric divisions, leading to an increase in CSCs over time. Treatment of CSCs with CBP/beta-catenin antagonists (e.g., ICG-001, C82) induces symmetric differentiative divisions within the CSC population, thereby eventually clearing the CSC population from the niche (**right panel**). Normal SSCs continue to undergo asymmetric divisions upon treatment with CBP/beta-catenin antagonists and are thus not depleted.

**Figure 4:**
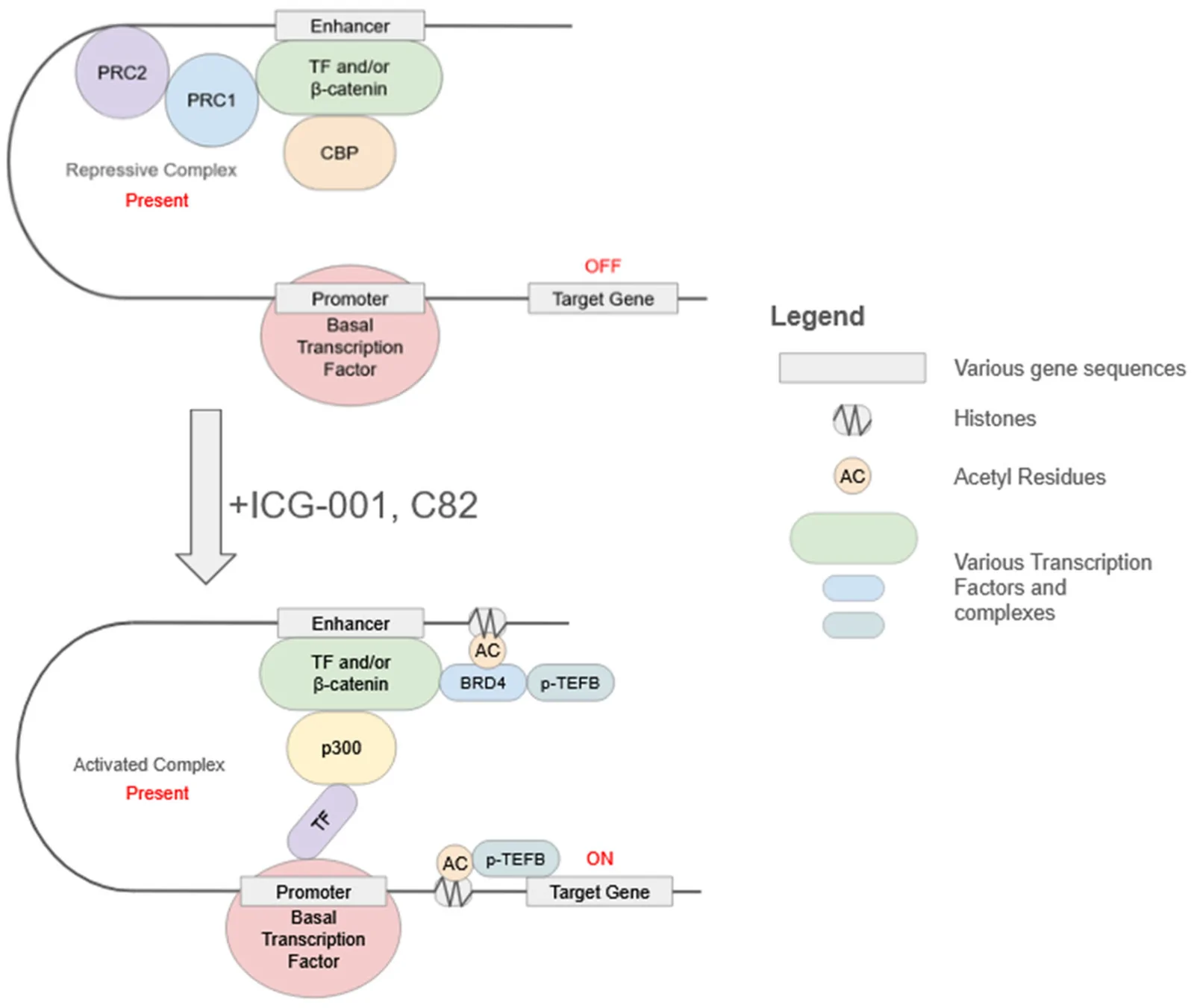
Intrinsically disordered regions (IDRs) of β-catenin connect transcription factor (TF)-interacting domains with the N-termini of either CBP or p300 in enhancer (E) and super-enhancer (SE) loci to interpret extracellular information and complex signaling cascades to orchestrate cell-specific responses. The model depicts a small molecule-specific CBP/β-catenin antagonist (ICG-001 or C82) dismissing CBP occupancy from a PRC1/PRC2 repressive complex with recruitment of p300 enhancing the assembly of tissue-specific E/SE with pleiotropic effects on differentiation, lineage identity and fidelity, coupled to cellular metabolism.

Can we safely correct the enhanced CBP/β-catenin-driven transcription associated with aging and diseases of aging? Although they possess extremely high biochemical specificity for the N-terminus of CBP, small molecule CBP/β-catenin antagonists possess highly pleiotropic activity due to their effects on E and SE [1,3,61,62,63]. Yet they have proven to be extremely safe both preclinically and clinically [3,64,65]. In IND enabling toxicology studies, the non-adverse event level for PRI-724 was 120 mg/kg/day in dogs given 28-day continuous infusion. Plasma concentrations were maintained at roughly 300 times the IC_50_ for 28 days. Furthermore, PRI-724 exhibited no dose-limiting toxicities, with dose escalation from 40 to 1280 mg/m2/day with 7 days of continuous i.v. infusion in cancer patients [64]. On-target efficacy was demonstrated via downregulation of the expression of the biomarker *survivin/BIRC5* with upregulation of the differentiation antigen *CK20*, in EpCAM^+^ selected circulating tumor cells (CTCs), strongly correlated with increasing plasma concentrations of drug (R = 0.97) [3,64]. CTCs provide a non-invasive, snapshot of tumor heterogeneity, metastasis, and therapy resistance, although only a small subpopulation of CTCs possess high tumor-initiating CSC capacity [66]. CBP/β-catenin antagonists via enhanced asymmetric differentiation of SSCs accelerate repair via activation without depletion of asymmetrically dividing SSCs [3,67,68]. Furthermore, taking advantage of the cell intrinsic preference of CSCs to divide symmetrically [25,26], CBP/β-catenin antagonists can force the activation of quiescent CSCs without changing the inherent preference of CSCs to divide symmetrically, thereby leading to their differentiation and the stochastic elimination of CSCs, or even pre-CSC, via symmetric differentiative division (Figure 3) [1,3,69,70,71,72]. Interestingly, it has been recently demonstrated that elimination of *Birc5*/*survivin* in oncogene-expressing stem cells prevents basal cell carcinoma (BCC) formation in mice [73]. The expression of survivin/*BIRC5* in CTCs was dose-dependently downregulated by the CBP/β-catenin antagonist PRI-724, implying that CBP/β-catenin antagonists potentially could be used to prevent cancer [3,64]. CBP/β-catenin antagonists assist in the maintenance of our SSCs via the regulation of mitochondrial function and metabolism involved in quiescence, differentiation and immune cell function [49,50,74]. In aged SSCs (as well as quiescent CSCs), CBP is apparently more likely to be found in repressive PRC1/2 complexes [75,76], preventing the timely activation of these quiescent stem cell populations, leading to ineffective homeostasis and repair (in the case of SSCs) or long-term tumor latency (in the case of CSCs). CBP/β-catenin antagonists can disrupt these repressive complexes to either enhance homeostasis and repair via asymmetric divisions (in the case of SSCs) or stochastically eliminate CSCs via symmetric differentiative divisions (Figure 4). By reestablishing the equilibrium between CBP/β-catenin versus p300/β-catenin-dependent transcription, which is corrupted with aging, small molecule CBP/β-catenin antagonists could potentially be safely used to treat or prevent many diseases of aging [3]. The development of next-generation highly specific, orally available CBP/β-catenin antagonists will allow for the testing of this concept in the clinic [3,77].

## Data Availability

This study did not report any previously unpublished data.
